# Bioinspired strontium magnesium phosphate cement prepared utilizing the precursor method for bone tissue engineering

**DOI:** 10.3389/fbioe.2023.1142095

**Published:** 2023-02-03

**Authors:** Qiaoyun Liu, Changjiang Liu, Weixing Wang, Liangjie Yuan, Yu Wang, Xinzeyu Yi, Zhenyu Pan, Aixi Yu

**Affiliations:** ^1^ Department of Orthopedics Trauma and Microsurgery, Zhongnan Hospital of Wuhan University, Wuhan, Hubei, China; ^2^ College of Chemistry and Molecular Sciences, Wuhan University, Wuhan, Hubei, China; ^3^ 16th Department, Plastic Surgery Hospital, Chinese Academy of Medical Sciences, Peking Union Medical College, Beijing, China

**Keywords:** bioinspired, precursor method, strontium magnesium phosphate, bone tissue engineering, biological activity

## Abstract

Bioinspired strontium magnesium phosphate cements for bone tissue engineering were prepared using a new, facile, environmentally friendly and high yielding (98.5%) precursor method. The bioinspired SMPCs have uniform particle distributions, excellent mechanical strengths and high biocompatibilities. The *in vitro* responses of bone marrow stromal cells to the SMPCs, including viability, osteogenic differentiation and alkaline phosphatase activity, were evaluated. The results show that the SMPC containing 0.5 mol of strontium (referred to as SMPC-2) has a higher degradation rate and biological activity than magnesium phosphate cements and the other SMPCs. In addition, the synergistic effect of strontium and magnesium ion release from SMPC-2 creates a conducive environment for cell proliferation, mineralized calcium deposition and new bone formation. These observations demonstrate the feasibility of using the new precursor method to generate SMPCs and the utility of these biologically compatible and highly effective cements for bone tissue engineering.

## Graphical Abstract

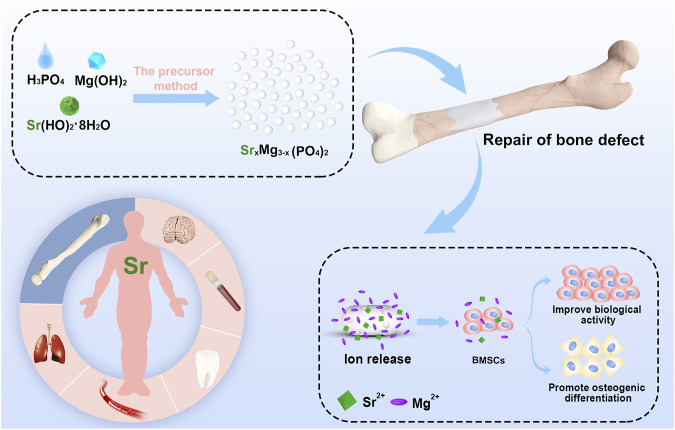

## 1 Introduction

Most of bone defects caused by trauma and tumor do not readily heal, and as a result, formation of bone non-union occurs (Dabiri et al., 2019). An effective method to rapidly restore the physiological function of defective bone tissue involves the use of appropriate bone graft materials (Pina et al., 2015; Nabiyouni et al., 2018). Inspired by the unique components of natural bones, magnesium phosphate cement (MPC) has been developed as a promising material for bone tissue engineering owing to its high mechanical strength, rapid curing propensity and good degradability (Babaie et al., 2017; Kanter et al., 2018; Yang et al., 2019). However, MPC is usually prepared by traditional methods involving hydration reactions of magnesium oxide and phosphate salts. A cement formed in this manner typically has a very short setting time and poor biocompatibility (Qiao et al., 2012). Consequently, methods for preparation of MPC that have practical clinical applications need to be devised. In one effort aimed at this goal, Yu et al. observed that the compressive strength of MPC rolled (23–58 MPa) and setting time (12–16 min) can be controlled by including Ca(H_2_PO_4_)_2_·H_2_O in the reaction mixture (Yu et al., 2019). Also, Wang et al. observed that the injectability and biocompatibility of MPC can be regulated by addition of citric acid (Wang et al., 2019). Moreover, Zhao et al. found that inclusion of gelatin microspheres (GM) and Ca(H_2_PO_4_)_2_·H_2_O improves the physicochemical, biodegradation and biocompatibility properties of MPC (Zhao et al., 2021).

Strontium (Sr) and magnesium belong to the alkaline earth metal family. As a trace element in human body, Sr not only promotes absorption of mineralized bone tissue by osteoclasts, it also causes an increase in the number of osteoblasts and induces osteoblasts to form bone matrix that leads to mineralized bone tissue generation (Arepalli et al., 2016; Liu et al., 2016; Kargozar et al., 2017). Early studies have shown that strontium has a dose-dependent effect on bone, which at certain dose levels can be beneficial to bones. Schorr et al. found that strontium promotes an increase of vertebral bone mass in patients with osteoporosis (Shorr and Carter, 1952). However, when strontium concentrations exceed the ideal levels, an adverse effect can take place on mineralization in the bone reconstruction process. For example, using histological and radiological methods, Matsunoto et al. found that administration of high-doses of strontium to rat tibia delays bone growth and the rate of endochondral ossification lysis (Matsumoto, 1976).

Based on these observations, it is reasonable to expect that by adding an appropriate amount of strontium the osteogenesis and repair of bone defects by MPC would be improved. At present, limited numbers of research efforts have focused on the bone repair properties of strontium doped MPCs and their clinical applications. In one investigation, He et al. observed that strontium modified magnesium phosphate bioceramics can be fabricated by employing a high-temperature (1,200°C) solid-state method and confirmed that this bone repair material inhibits the formation of osteoclasts (He et al., 2019). However, the material synthesized in this way had a fixed shape so that it was only suitable for repairing defects in bones having the same shape.

In the current study, a precursor method was developed for large scale synthesis of bioinspired strontium doped magnesium phosphate cement (SMPC) for use in bone tissue engineering. This method is widely used in the preparation of materials for lithium-ion batteries because it is both green and controllable (Xie et al., 2019; Liu et al., 2021). The precursor (P-SMP) used in this approach is generated by mixing magnesium hydroxide, strontium hydroxide with phosphoric acid. P-SMP has small particle sizes, uniformly distributed components, a large surface energy, and high activity and biocompatibility. The precursor method, which involves heating the P-SMPs to 800°C followed by mixing the formed strontium doped magnesium phosphates (SMPs) with KH_2_PO_4_ and MgO. Preparation of the cements has a low energy consumption and high yield, both of which make it suitable for large-scale synthesis. In addition, the SPMCs generated in this manner have a suitable setting time at room temperature and they can be arbitrarily shaped to meet the varying needs of bone defect patients. In addition, effects of using different amounts of Sr on the properties of the SMPCs were explored and a survey was made of key biological properties of this cement. The results of this investigation should promote interest in practical use of SMPC in bone repair engineering.

## 2 Materials and methods

### 2.1 Materials

Mg(OH)_2_ (AR) and Sr(OH)_2_·8H_2_O (AR) were purchased from Aladdin Inc., China. H_3_PO_4_ (AR, ≥85 wt%.), (MgCO_3_)_4_·Mg(OH)_2_·5H_2_O (AR) and KH_2_PO_4_ (AR) were purchased from Sinopharm Chemical Reagent Co., Ltd. All chemicals were used as received.

### 2.2 Preparation of bioinspired MPC and SMPCs

#### 2.2.1 Preparation of the precursors P-MP and P-SMPs

To 800 g of stirred water (200 rpm) was added 174 g of Mg(OH)_2_. After stirring for about 5 min the magnesium hydroxide became evenly dispersed in the water. Following increasing the stirring speed to 300 rpm, 230.6 g of H_3_PO_4_ was added dropwise to the solution over a 10 min period. Stirring was continued for 10 min, at which time the pH of the solution was 7.5. The formed solid was separated by filtration and the formed filter cake was dried for 10 h at 100°C to give the precursor P-MP. The same procedure was utilized to prepare the Sr containing precursors P-SMP-1, P-SMP-2, P-SMP-3 and P-SMP-4 with the exception that 0.25, 0.5, 0.75 and 1.0 mol, respectively, of Sr(OH)_2_·8H_2_O were used in place of Mg(OH)_2_.

#### 2.2.2 Preparation of MP and SMPs

P-MP was heated in a muffle furnace to 800°C at a rate of 3°C/min and then at 800°C for 2 h to yield MP. The SMPs, SMP-1, SMP-2, SMP-3 and SMP-4 were generated using the same heat treatment method. The contents of these materials are given in Table 1.

**TABLE 1:** Contents of MP and SMP.

Sample	Content of Sr/moL	Composition	Theoretical weight/g	Yield/g	% yield
MP	0	Mg_3_(PO_4_)_2_	262.86	259.65	98.78
SMP-1	0.25	Mg_2.75_Sr_0.25_(PO_4_)_2_	278.69	274.87	98.63
SMP-2	0.5	Mg_2.5_Sr_0.5_(PO_4_)_2_	294.55	290.74	98.71
SMP-3	0.75	Mg_2.25_Sr_0.75_(PO_4_)_2_	310.34	307.31	99.02
SMP-4	1.0	Mg_2_Sr(PO_4_)_2_	326.17	321.29	98.50

#### 2.2.3 Preparation of bioinspired MPC and SMPCs

MgO was generated by sintering magnesium carbonate ((MgCO_3_)_4_·Mg(OH)_2_·5H_2_O) at 800°C for 2 h. MP or the SMPs, KH_2_PO_4_ and MgO were mixed in the mass ratio of 2.62:4.08:0.80 at room temperature. Addition of deionized water in the mass ratio of 1.5:1 and standing for 2 min led to production of MPC or the SMPCs. Cylindrical samples of these materials, obtained letting each cement to stand in a polytetrafluoroethylene cylindrical mold (6 mm diameter, 12 mm height) for 7 d at 37°C and 100% humidity, were subjected to strength tests. Cements with dimensions of 6 mm diameter and 2 mm height were subjected to disinfection under high temperature and pressure, and then used to assess cell viability, *in vitro* degradation performance, alkaline phosphatase (ALP) activity, and for alizarin Red S and cell living/death staining.

### 2.3 Characterization

All materials prepared in this effort were analyzed using thermogravimetry (TG) with a Netzsch STA449 thermal analyzer (Netzsch, Germany) at a heating rate of 10°C/min in flowing air. P-MP, P-SMP, MP, SMP, MPC and SMPC were also studied using X-ray diffractometry (XRD), to identify their crystalline phases. XRD measurements were carried out using a Rigaku Miniflex600 diffractometer (Rigaku, Japan) with Cu Ka radiation (k = 1.54178 Å) at a scanning rate of 6°/min. Registrations were performed in the 10°–80°, 2(θ) range. To determine morphologies, and particle sizes and distributions, scanning election microscope (SEM) images were recorded using a JSM-6510 scanning election microscope (JEOL, Japan).

### 2.4 Setting time and compressive strength

The setting time was measured using a Vicat instrument, which was defined as the time taken before the needle cannot penetrate the sample by 1 mm. Compressive strengths of the materials were measured at a loading rate of 1 mm/min using a universal testing machine (Instron 5,967, United States) according to BS EN 12390–3:2009 (British Standard Institution, 2009). Five replicates of each measurement were made.

### 2.5 pH variation and cement degradation

In triplicate measurements, the initial weights (W_0_, based on solid/liquid mass ratio of 0.2 g/g) of disc samples of each material were recorded and then each disc is immersed in oscillating (100 rpm) Tris HCl buffer (pH = 7.4) at 37°C. Tris HCl buffer was refreshed once a day in the first week and once a week later. Disc samples were removed on the 1st, 3rd, 7th, 14th, 21st and 28th day, and the pH of the Tris HCl buffer was determined (FE-28, Mettler, China). The removed disc samples were rinsed with deionized water, dried at 80°C for 2 h, and then weighed (W_t_). Percent weight loss was calculated using following Eq. 1.

$$Weight\;loss\;\left(\%\right)=\frac{W_{0}-W_{t}}{W_{0}}\times 100\%$$ (1)

### 2.6 Mg^2+^and Sr^2+^ release

In triplicate experiments, discs in Tris HCl buffer, produced in the above manner, were removed on the 1st, 3rd, 7th, 14th, 21st and 28th d to give remaining clear liquids whose Mg^2+^ and Sr^2+^ concentrations were determined using inductively coupled plasma mass spectrometery (QP-MS, Jena AG, Germany).

### 2.7 Extract solutions and cell culture

Each disc sample was placed in complete medium (from Procell). The resulting system was oscillated at 100 rpm and 37°C for 24 h (Wang et al., 2019). The complete medium (referred to as extracted complete medium) was collected for later cell culture experiments. In a separate experiment, complete medium containing the discs were replaced by osteogenic inducing medium, the extracted osteogenic inducing medium (extracted OIM) was used to study osteogenic induced differentiation.

Rat mesenchymal bone marrow stem cells (BMSCs) (from Procell) were cultured under 5% CO_2_ in complete medium at 37°C. The extracted complete medium was replaced every third day. In the study of osteogenic differentiation, after the BMSCs culturing at 37°C under 5% CO_2_ for 1 d, complete medium was replaced by extracted complete medium. When the density of the BMSCs reached 90%, the complete medium was replaced by extracted OIM which renewed every third day.

### 2.8 Cytotoxicity analysis

The *in vitro* cytotoxicity of disc samples of each material was evaluated in quintuplicate using a CCK-8 assay. BMSCs in complete medium were seeded in 96 well plates at a density of 1 × 10^5^ cells/well, and then cultured at 37°C under 5% CO_2_ for 1 d. Complete medium was replaced by extracted complete medium and complete medium was used as control. After culturing for 1, 3 and 5 d, extracted complete medium in each well was removed and replaced by 90 μL complete culture medium containing 10 μL CCK-8 reagent. After 2 h incubation, the optical density (OD) at 450 nm in each well was determined using a microplate reader (FC, ThermoFisher, United States). Cell viability was calculated using the following Eq. 2.

$$Cell\;viability\;\left(\%\right)=\frac{OD\;\left(dose\right)-OD\;\left(blank\right)}{OD\;\left(0\;dose\right)-OD\;\left(blank\right)}\times 100\%$$ (2)

### 2.9 Live/dead cell ratio

BMSCs in complete medium were seeded in 24 well plates at a density of 5 × 10^5^ cells/well, and then cultured at 37°C with 5% CO_2_ for 1 d. Complete medium was replaced by extracted complete medium and then after 5 d the extracted complete culture medium was removed. According to the procedure provided by the kit manufacturer, 150 μL of a solution of 4′,6-diamidino-2-phenylindole (dead cells: red fluorescence light) and calcein acetoxymethyl ester (live cells: green fluorescence light) was added to each well, which were incubated for 30 min. Distributions of live/dead cells were determined by using a fluorescence microscope (ICX41, SOPTOP, China).

### 2.10 Alkaline phosphatase activity

The activity of alkaline phosphatase (ALP) was analyzed determined using the Alkaline Phosphatase Assay Kit (Beyotime, China). BMSCs in complete medium were seeded in 24 well plates at a density of 5 × 10^5^ cells/well and then cultured at 37°C with 5% CO_2_ for 1 d. Complete medium was replaced by extracted OIM, which was removed after BMSCs culturing for 7 and 14 d. According to the procedure provided by the kit’s manufacturer, 50 μL western and IP cell lysate was added to each well for 1–2 min. The lysates were mixed with *p*-nitrophenyl phosphate (PNPP) and incubated for 30 min. Optical densities (OD) at 405 nm were determined using a microplate reader (FC, ThermoFisher, United States).

### 2.11 Alizarin red S staining

BMSCs in complete medium were seeded in 24 well plates at a density of 5 × 10^5^ cells/well, and then cultured at 37°C with 5% CO_2_ for 1 d. Complete medium was replaced by extracted OIM. After 14 and 28 d, extracted OIM was removed, and the cells were treated with 4% neutral formaldehyde for 30 min, placed in an Alizarin red staining solution for 30 min and washed twice with PBS. Analysis of the stained cells was accomplished using a microscope (ICX41, SOPTOP, China).

### 2.12 Statistical analysis

All data is presented as means ± standard deviation. Data from each group were analyzed by using GraphPad Prism eight statistical software using one-way analysis of variance (ANOVA). When *p*-value <0.05, the data were considered had statistical difference.

## 3 Results and discussion

### 3.1 Synthesis and characterization

**FIGURE 1:**
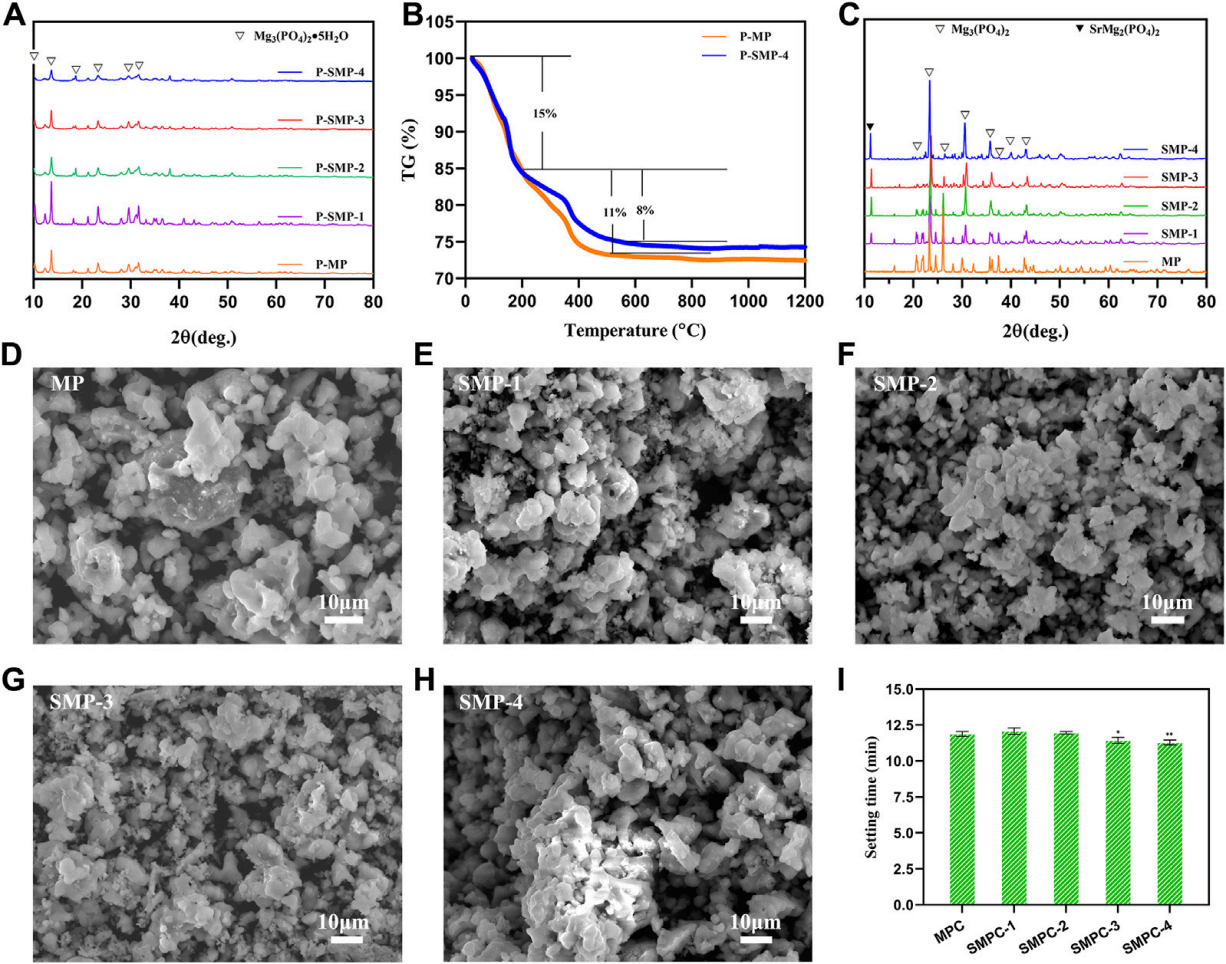
Materials characterization. **(A)** The XRD patterns of precursor P-MP and the P-SMPs. **(B)** TG curves of P-MP and P-SMP-4. **(C)** XRD patterns of MP and SMP. **(D–H)** SEM morphologies of the MP and the SMPs. **(I)** Setting time of the cements MPC and the SMPCs; the data are presented as mean ± SD (*p <0.05; **p <0.01; vs. MPC).

Analysis of the XRD patterns (Figure 1A) shows that the major components in P-MP and P-SMP is Mg_3_(PO_4_)_2_·5H_2_O (PDF# 35–0329), and that as the amount of strontium in the P-SMPs increases the degree of crystallinity becomes lower. Also, the presence of strontium does not affect the crystalline phases of the P-SMPs, likely because at room temperature used for their preparation strontium exists as an amorphous substance.

In Figure 1B are displayed TG curves of P-MP and P-SMP-4. The weight loss profiles of these precursors are similar and that thermal decomposition of these materials takes place in two steps. The first step, corresponding to about a 15% weight loss, occurs from 25°C to 200°C, which is associated with volatilization of water from Mg_3_(PO_4_)_2_·5H_2_O. The second step takes place from 200°C to 550°C and it corresponds to respective weight losses form P-MP and P-SMP-4 of about 11% and 8%. This step is associated with decomposition of the amorphous component in each precursor. No weight changes occur above 550°C as is expected for the produced MP and SMP. Based on these results, combustion temperatures of above 200°C, can possibly be used for the heat treatment of P-MP and P-SMP. Considering other factors including compressive strength and setting time, 800°C for 2 h is preferrable for generating MP and SMP from the respective precursors.

X-ray diffraction analysis was performed to investigate the effects of strontium on the crystalline phases of the SMPs (Figure 1C). The pattern of pristine MP is completely consistent with the one in the database for Mg_3_(PO_4_)_2_ (PDF# 35–0329). As the content of Sr in the SMPs.

Increases, the XRD pattern begins to increasingly include peaks associated with SrMg_2_(PO_4_)_2_ (PDF# 52–1,590). In addition, no other impurity peaks derived from impurities are present in the XRD patterns. The results demonstrate that MP and the SMPs, prepared using the precursor method, have high crystallinities and purities. In addition, the precursor method is simple, environmentally friendly, and high yielding (98.50%–99.02%) (Table 1).

The morphologies of MP and the SMPs were assessed using scanning electron microscopy (Figures 1D–H). The results show that the basic morphology of MP remains unchanged when strontium is included, but increases in Sr content leads to creation of more uniformly distributed small particles with sizes less than 4 μm. In particular, the particle distribution in SMP-2 is more uniform, and 50% of the particles have sizes less than 2 μm, which may be conducive to the release of Sr^2+^ during the *in vitro* degradation (Arepalli et al., 2016). The setting time of the cements, MPC and the SMPCs was measured (Figure 1I). The results show that the setting time of MP is about 12.08 min, and that the setting time of SMPCs decreased slightly with Sr content, but the difference was not significant. The setting time of MPC and SMPCs ranged from 11.23 min to 12.08 min, meeting the requirement of clinical bone repair materials for setting time of 8–15 min (Khairoun et al., 1998).

**FIGURE 2:**
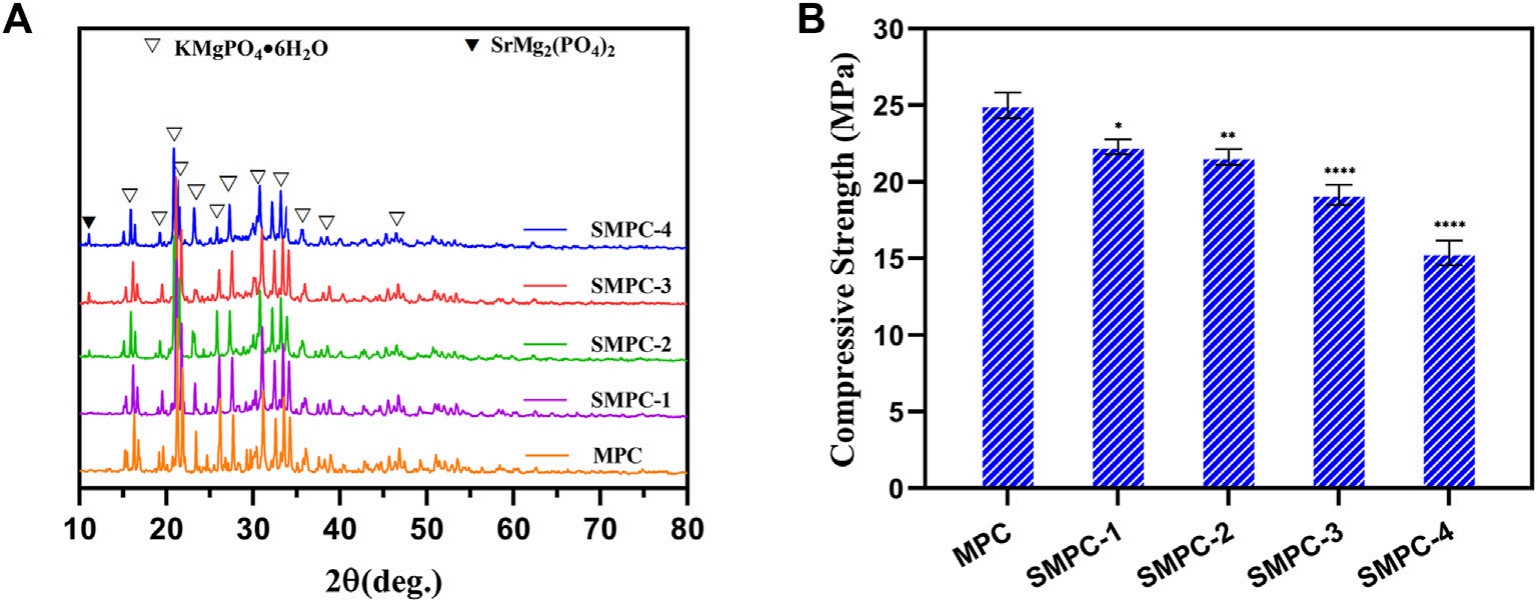
**(A)** XRD patterns of the MPC and SMPCs. **(B)** Compressive strength of the cements MPC and the SMPCs; the data are presented as mean ± SD (*p <0.05; **p <0.01; ****p <0.0001; vs. MPC).

XRD patterns of cements, MPC and the SMPCs, cured at 37°C for 7 d are displayed in Figure 2A. It should be noted that because the hydration product of MPC is KMgPO_4_·6H_2_O the XRD peaks of MPC correspond to those of the mixed K and Mg containing phosphate (PDF# 35–0812). The presence of Sr does not affect the crystallinity of the SMPC cements. Moreover, SMP does not become hydrated during the process of bone cement formation so that XRD spectra of the SPMCs contain peaks associated with both KMgPO_4_·6H_2_O and SrMg_2_(PO_4_)_2_. Also, analysis of the XRD patterns in Figure 2A indicates that unreacted MgO does not remain in MPC and SMPC after bone cement formation. As a result, these cements should have better biocompatibility than MPC prepared from dead burned MgO (Mestres and Ginebra, 2011; Qiao et al., 2012). The compressive strengths of the cements, MPC and the SMPCs were determined after curing at 37°C for 7 d (Figure 2B). The results show that the compressive strength of MP is about 25 MPa, and that the compressive strength decreases as the amount of strontium increases from 0.25 mol to 1.0 mol. Importantly, when the strontium content is 0.5 mol (SMPC-2), the compressive strength is 22.5 MPa, which meets the requirements for bone repair materials. The compressive strength changes likely result from the fact that the radius of Sr^2+^ is slightly larger than that of Mg^2+^, and that this difference causes a change in the lattice structure of the MPC.

### 3.2 *In vitro* degradability and cytotoxicity of MPC and the SMPCs

**FIGURE 3:**
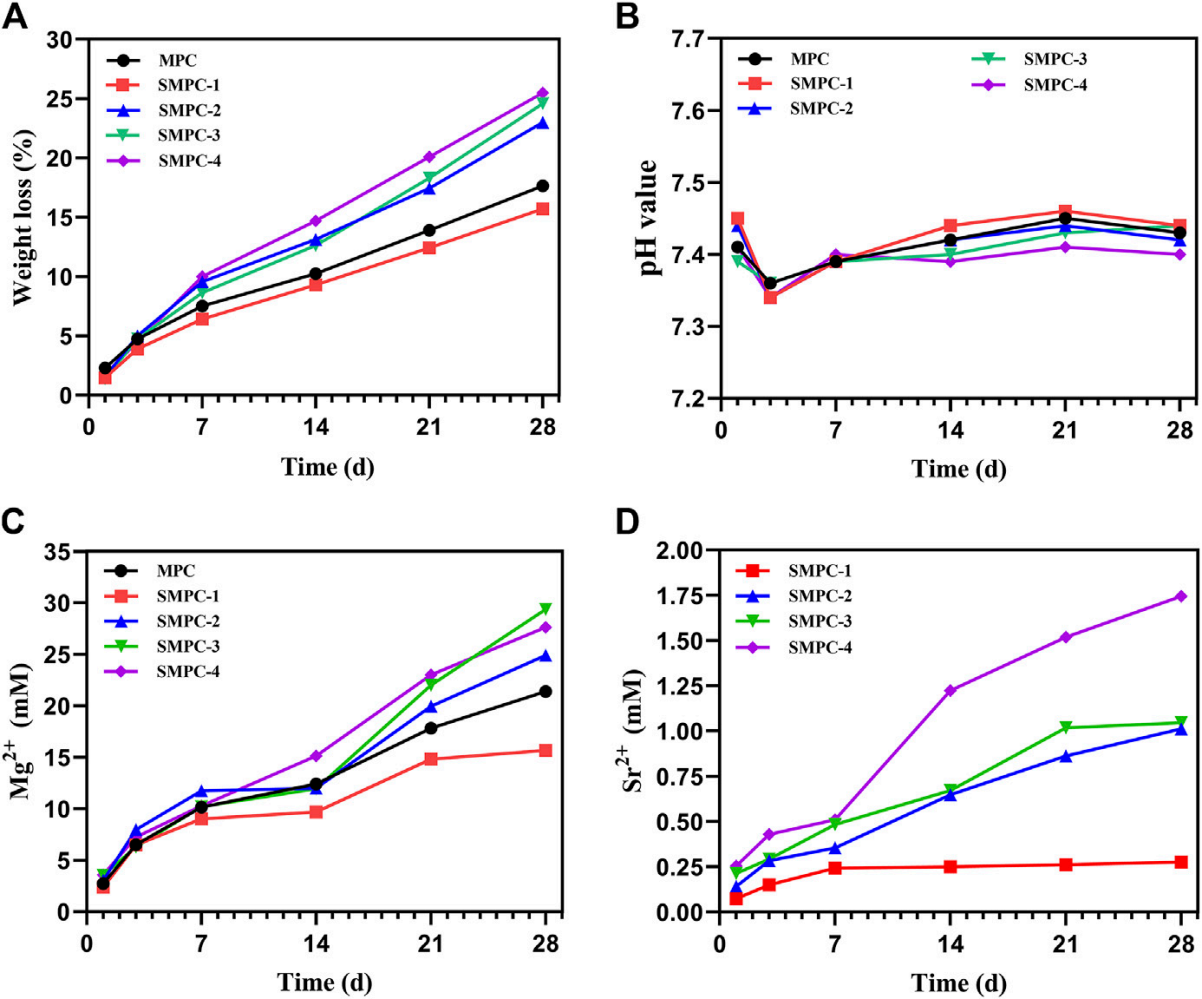
*In vitro* degradation propensities of MPC and the SMPCs soaking in Tris HCl (pH = 7.4) buffer: **(A)** Weight loss of the MCP and SMPC cements. **(B)** The pH of the MCP and SMPCs. **(C)** Mg^2+^ cumulative content released from MCP and SMPCs. **(D)** Sr^2+^ cumulative content released from SMPCs.

Biodegradability is critical requirement of artificial bone repair materials, because it leads to cell infiltration and better *in vivo* absorption (Zhai et al., 2018). Thus, the *in vitro* biodegradation properties of MPC and the SMPCs were evaluated. In Figures 3A, B are plots of the respective time dependencies of weight loss percentages and pH occurring upon immersing the bone cements in Tris HCl (pH = 7.4) buffer for 1, 3, 7, 14, 21 and 28 d. The *in vitro* degradation trends

Of all cements are similar and degradation in the first week is significantly larger than those occurring during longer time periods (Figure 3A). The total weight loss of MPC is 17.6% after 28 d of the *in vitro* degradation. When the strontium content is 0.25 mol (SMPC-1), the rate of *in vitro* degradation is slightly lower that of MPC. When the amount of strontium is 1.0 mol (SMPC-4), *in vitro* degradation after 28 d is 25.5%, suggesting that the biodegradability of bone cement is strongly promoted by strontium. The high *in vitro* degradation performance is likely caused by the replacement of magnesium by strontium, which weakens the crystal structure that is conducive to the release of metal ions.

As shown in Figure 3B, the pH values of media containing MPC and the SMPCs decrease slightly during the 1^st^-3^rd^ day period but then undergo slight increases as the soaking time is extended. During the soaking periods, pH values of media containing MPC and the SMPCs are similar in the 7.35–7.45 range that is conducive to cell growth. In a previous study, a solid-phase method utilizing excess MgO was employed to prepare MPC at a high temperature. This approach generates a cement whose degradation creates an alkaline environment (pH > 7.5), which has an adverse effect on cell growth (Qiao et al., 2012). In the newly developed precursor method, only 6.4% of active MgO is used for cement formation considering mechanical strength and setting time and as a result, no unreacted MgO remains, as demonstrated by the results of XRD analysis (Figure 2).

To investigate metal ion release from MPC and SMPCs during under cell culture conditions, the contents of Mg^2+^ and Sr^2+^ in the Tris HCl solution after 1–28 d were determined using inductively coupled plasma mass spectrometery. (Figures 3C, D). The rate of release of Mg^2+^ from MPC in the first 7 d period is significantly higher than it is during the later time periods (Figure 3C). The content of Mg^2+^ in the medium arising from soaking MPC for 28 d is 15.66 mM. Interestingly, the rate of release of Mg^2+^ from all SMPCs except SMPC-1 increases with Sr content. This phenomenon could be associated with weakening of lattice structure brought about by replacement of Mg^2+^ by Sr^2+^. By inspection of Figure 3D it can be seen that the rate of Sr^2+^ release from the SMPCs also increases as the Sr content increases and that the total amount of Sr^2+^ released from SMPC-2 after 28 d is 0.14–1.01 mM. The results are consistent with those arising from the *in vitro* degradation studies (Figure 3A). Based on the above results, it can be concluded that the SMPCs prepared using the precursor method display good degradation performances *in vitro* and provide a proper environment for cell growth.

**FIGURE 4:**
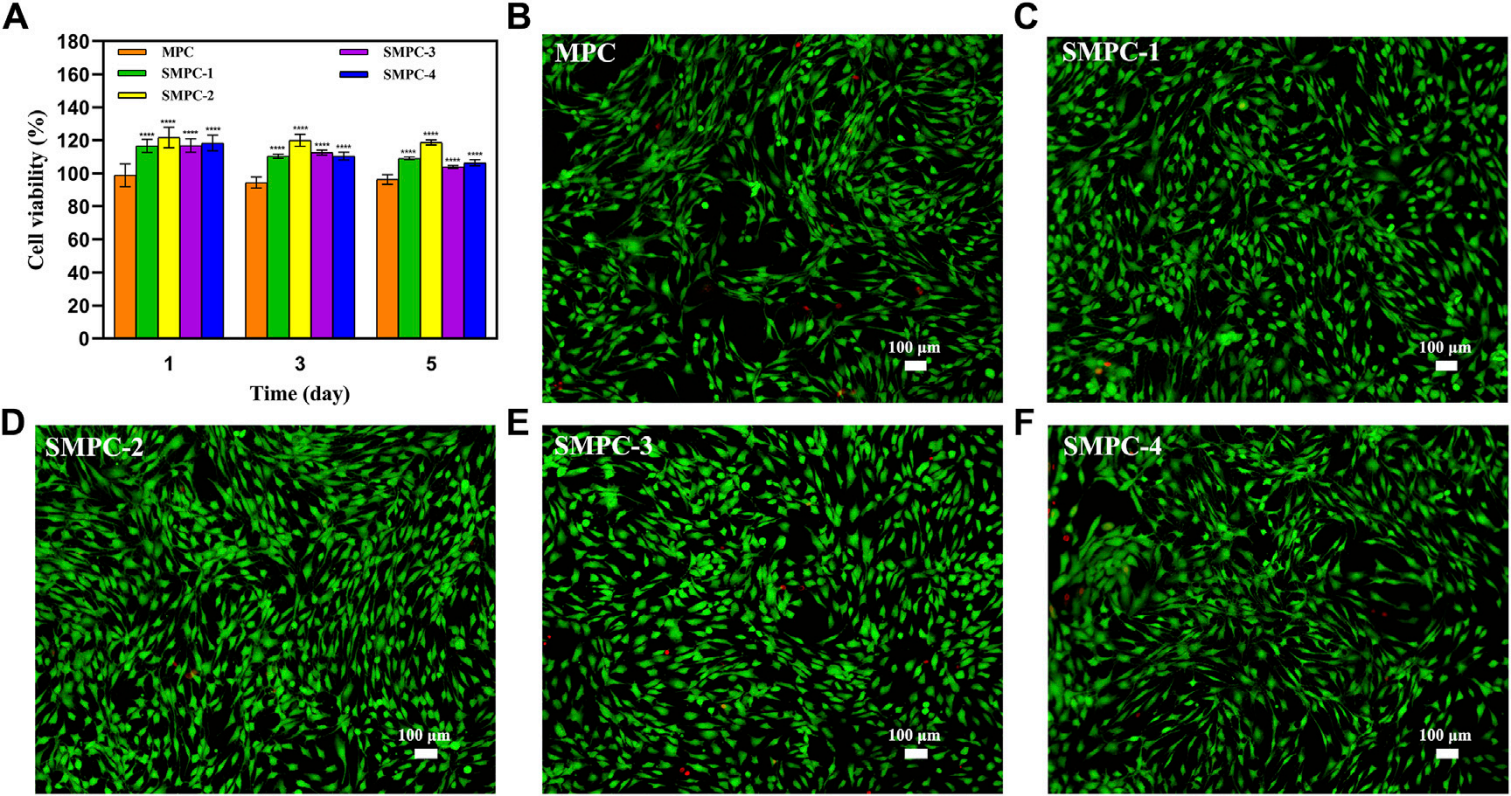
**(A)** Results of CCK-8 assays of BMSC bioactivities in the presence of MPC and the SMPCs after culturing for 1, 3, and 5 d. Data are presented as mean ± SD (****p <0.0001; vs. MPC). **(B–F)** Fluorescence microscope images of 4′,6-diamidino-2-phenylindole and calcein acetoxymethyl ester stained BMSCs cultured in the presence of MPC and the SMPCs for 5 d (green: viable cells; red: dead cells).

The CCK-8 assay can be employed to evaluate the effects of MPC and the SMPCs on BMSC viability. The results (Figure 4A) show that activities of the of SMPC treated cells over a 1–5 d period are larger than those of cells treated with MPC, suggesting that Sr2^+^ promotes cell proliferation. For example, the activity of SMPC-2 treated cells is 120% during the 3 d period, indicating that it has the highest biocompatibility. This finding suggests that a cooperative effect occurs at Sr2^+^ concentrations of 0.14–1.01 mM and Mg2^+^ concentrations of 2.74–21.39 mM to enhance the synthesis of intracellular proteins and DNA, which are considered to be key regulators of cell proliferation and differentiation (Rubin, 1975).

For evaluation of the effects of MPC and the SMPCs on the live/dead cell ratio, fluorescence microscope images of 4′,6-diamidino-2-phenylindole and calcein acetoxymethyl ester stained BMSC were obtained after a 5 d culture period (Figures 4B–F). Inspection of the images shows that when SMPCs are present in the incubation mixture, the density of live BMSCs is significantly enhanced relative to that for cells grown in the presence of MPC. In addition, only a small number of dead cells form in the SMPCs treated mixture. Moreover, SMPC-2 (0.5 mol Sr) promotes formation of the greatest number of viable BMSCs and lowest number of dead cells. The results show that a defined amount of Sr in the cement (e.g., SMPC-2) leads to an improvement of cell viability, which is consistent with the CCK-8 assay results in Figure 4A. Overall, these experiments suggest that SMPCs prepared using the precursor method more potently promote cell growth and proliferation.

### 3.3 Osteogenic differentiation

**FIGURE 5:**
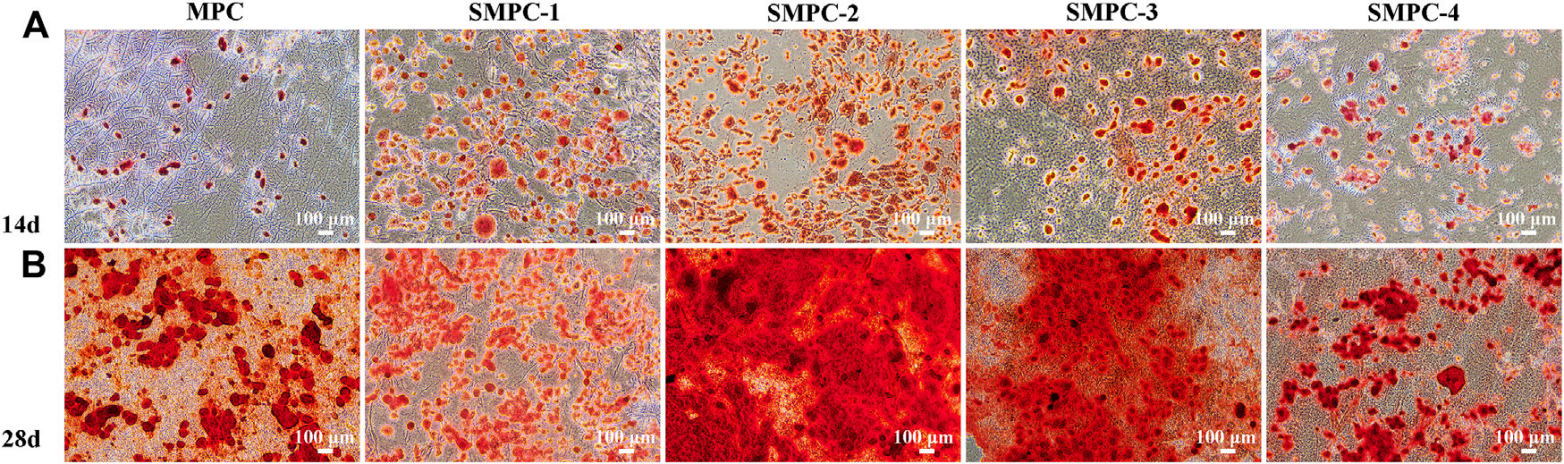
BMSCs stained with alizarin red S solution after culturing 14 d **(A)** and 28 d **(B)** in the presence of MPC and the SPMCs.

Mineralized calcium content is a key marker for osteoblast proliferation and differentiation, as well as the osteogenic potential of bone tissue. The results of osteoblast proliferation studies using the Alizarin Red S staining method are given in the form of microscope images of stained BMSCs in Figure 5. Alizarin red combines with calcium ions to form a stable red complex, which can detect the mineralization degree of osteoblasts. The adsorption amount of Alizarin red dye is in direct proportion to the calcium content (Wang et al., 2016). The results show that inclusion of Sr in the MPC cement used to treat these cells facilitates osteogenic induction and.

Differentiation after incubation for 14 and 28 d, and that the enhancement is dependent on the Sr content of the SMPCs and the incubation time. Specifically, after 14 d, the mineralization effect initially increases with Sr content, reaches a plateau at a 0.5 mol Sr (SMPC-2) and then decreases (Figure 5A), and in all cases the enhancement effects are greatly magnified at 28 d (Figure 5B). These observations are consistent with a previous study which showed that Sr alone induces bone marrow mesenchymal stem cells to osteogenesis (Arepalli et al., 2016).

**FIGURE 6:**
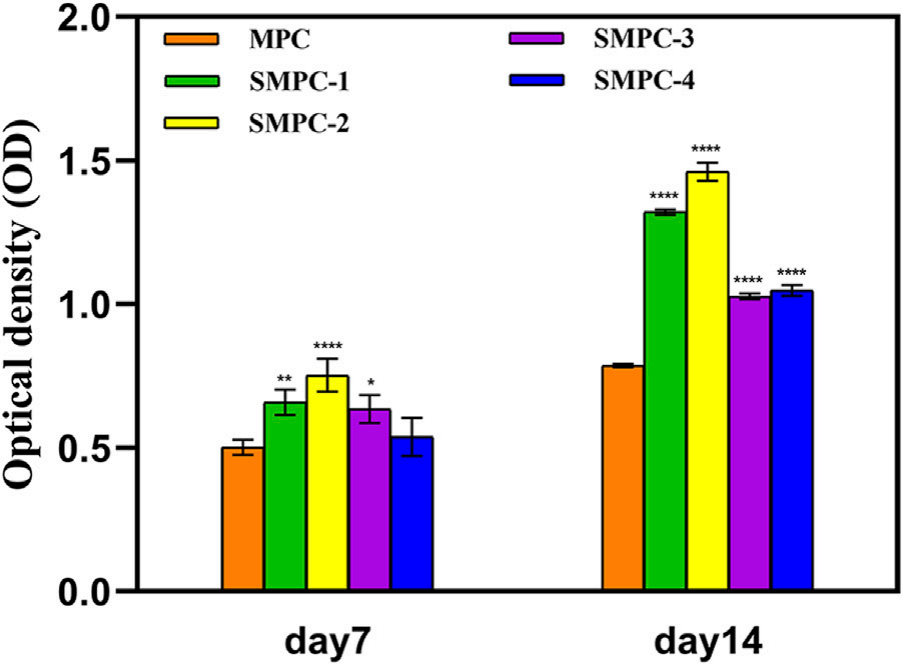
ALP activities in the form of optical densities in assays of BMSCs cultured for 7 and 14 d in the presence of MPC and the SPMCs. Data are presented as mean ± SD (*p <0.05; **p <0.01; ****p <0.0001; vs. MPC).

The content of alkaline phosphatase (ALP) directly reflects the activity of osteoblasts, which positively correlates with the mineralization ability of osteoblasts. The bar graphs in Figure 6 show alkaline phosphatase activities in BMSCs first increase and then decreases with increasing Sr content in the SMPCs. This finding is consistent with the mineralization results given in Figure 5, and the combined results indicate that SMPC-2 has the best osteogenic differentiation effect. It has been reported that when the Sr2^+^ concentration is in the 0.34–1.0 mM range, differentiation activity and osteoclast mediated resorption reduction of bone or bone substitutes takes place (Gentleman et al., 2010; Montesi et al., 2017). The observations definitively demonstrate that Mg^2+^ in the concentration range of 14.36–26.27 mM has an up-regulating effect on osteoblast proliferation (Wu et al., 2015), and inclusion of 0.14–1.01 mM Sr^2+^ further promotes osteogenic differentiation of stem cells (Gentleman et al., 2010).

## 4 Conclusion

In this study, bioinspired strontium magnesium phosphate cements (SMPCs) possessing various Sr contents (0–1.0 mol) were prepared by using a newly developed precursor method. The synthetic process utilizing this protocol is simple and environment-friendly, and high yielding (98.5%). The SMPCs were found to have and uniform particle distributions, and high crystallinities, purities and compressive strengths. Compared to MPC, the SMPCs have higher rates of biodegradation to form both free Sr2^+^ and Mg2^+^ and, as a result, they create a more favorable environment for growth of BMSCs. Particularly significant is the observation that shows that SMPC-2 containing 0.5 mol of Sr has the highest bioactivity. The synergistic effect of Sr2^+^ and Mg2^+^ release from SMPC-2 creates a more conducive environment for promoting cell proliferation, mineralized calcium deposition and bone tissue formation. These properties make the SMPCs promising cements for damaged bone repair applications. In the future, investigations will be carried out to improve the mechanical properties of the SMPCs, to enhance the understanding of the foundation of their *in vivo* repair effects, and finally to apply these cements in clinical environments.

## Data Availability

The raw data supporting the conclusions of this article will be made available by the authors, without undue reservation.
